# Intracranial aneurysm segmentation on digital subtraction angiography: a retrospective and multi-center study

**DOI:** 10.3389/fneur.2025.1646517

**Published:** 2025-10-13

**Authors:** Ruibo Liu, Ruixuan Zhang, Wei Qian, Guobiao Liang, Guangxin Chu, Hai Jin, Ligang Chen, Jing Li, He Ma

**Affiliations:** ^1^College of Medicine and Biological Information Engineering, Northeastern University, Shenyang, Liaoning, China; ^2^Department of Neurosurgery, General Hospital of Northern Theater Command, Shenyang, Liaoning, China; ^3^Department of Neurology, The Fourth Affiliated Hospital of China Medical University, Shenyang, Liaoning, China

**Keywords:** intracranial aneurysms, digital subtraction angiography, deep learning, edge-aware local attention, global shape-aware fusion block

## Abstract

**Introduction:**

Accurate segmentation of intracranial aneurysms (IAs) in digital subtraction angiography (DSA) is critical for endovascular embolization and risk assessment of ruptured IAs. However, this task remains challenging due to problems like vascular overlap, small target size and similarity to ring blood vessels. To develop a novel deep learning model to improve segmentation performance of IAs on DSA datassets, especially addressing challenges of small IAs.

**Methods:**

We propose a novel deep learning model, the Shape-aware dual-stream attention network (SDAN). This network integrates two novel modules: (1) Edge-aware Local Attention Module (ELAM), which differentiates aneurysms from adjacent vasculature by capturing morphological features, (2) Global Shape-aware Fusion Block (GSFB) that enhances pattern recognition through contextual aggregation between domains. The model was trained and tested on 62,187 retrospective DSA images from three institutions, with external validation on 26,415 images. Performance was evaluated using DSC, HD95, and sensitivity.

**Results:**

The proposed SDAN outperforms the other models when tested on multiple centers separately with an average Dice score of 0.951 on the internal test set and 0.944 on the external test set. We also evaluated the different sizes of aneurysms individually and the results show that SDAN outperforms the other models on all sizes of aneurysms. This study demonstrates the effectiveness of SDAN for intracranial aneurysm segmentation.

**Conclusion:**

Our proposed SDAN significantly improves the accurate segmentation of intracranial aneurysms in DSA images beyond existing medical image segmentation models. The model solves the problems of small intracranial aneurysms that are not easily segmented accurately, over-segmentation caused by the similarity of intracranial aneurysms and ring vessels, and under-segmentation caused by the overlap of neighboring vessels.

## 1 Introduction

Intracranial aneurysms (IAs) represent a critical cerebrovascular pathology, with rupture leading to aneurysmal subarachnoid hemorrhage (aSAH). This condition carries a devastating mortality rate exceeding 50% (1–3). Survivors frequently suffer severe neurological deficits, imposing significant burdens on global healthcare systems (4). Endovascular embolization has emerged as a primary therapeutic intervention; however, its efficacy critically depends on precise IA delineation for preoperative planning and intraoperative navigation (5, 6). Substantially, inaccurate segmentation may compromise the success of interventional treatments (such as endovascular coiling), thereby increasing the risk of perioperative complications like coil protrusion or incomplete occlusion (7). This highlights that sub-millimeter precision is essential in clinical workflows (8).

Current reliance on manual delineation by neurointerventionists is prohibitively slow (> 1.5 minutes per frame) and exhibits substantial variability, particularly for small aneurysms (diameter < 3*mm*) and in cases of vascular overlap or occlusion (9, 10). This inefficiency delays critical interventions and elevates patient risk during time-sensitive procedures, highlighting the urgency for automated segmentation solutions. Digital subtraction angiography (DSA) remains the gold standard for IA characterization due to its superior spatial resolution (150 − 200μ*m*/*pixel*) and clear depiction of vascular anatomy, making it indispensable for intraoperative guidance (11–13). In contrast, computed tomography angiography (CTA) and magnetic resonance angiography (MRA) are more widely adopted for screening owing to their noninvasive nature, with datasets often including patients without aneurysms to reflect real-world clinical scenarios (14, 15). However, these modalities are susceptible to motion artifacts and offer lower temporal resolution, limiting their utility in complex interventional settings.

The lack of segmentation tools that are accurate, capable of real-time operation, and sensitive to small IAs for single-frame 2D DSA represents a critical gap. Furthermore, the clinical translation of existing models is hindered by insufficient validation on large-scale, multi-center DSA datasets. To address these limitations, this study introduces the Shape-aware Dual-stream Attention Network (SDAN), explicitly designed for accurate IA segmentation. Our innovations include: (1) Edge-aware Local Attention Module (ELAM): A learnable edge detection module combining Canny operator with convolutions and local attention to capture morphological features, addressing the problem of distinguishing IAs from adjacent vasculature; (2) Global Shape-aware Fusion Block (GSFB): A cross-dimensional feature aggregator integrating spatial-channel attention mechanisms, enabling robust recognition through hemodynamic context modeling.

We trained and evaluated SDAN on a retrospective dataset comprising 62,187 DSA images from three independent institutions. Our objective is to deliver a clinically viable segmentation tool that significantly outperforms existing state-of-the-art methods, particularly for small IAs, thereby enabling faster, safer, and more precise endovascular interventions.

## 2 Related work

### 2.1 Clinical challenges in accurate segmentation of IAs

The segmentation of intracranial aneurysms (IAs) remains a high-stakes clinical challenge where computational precision directly affects procedural safety. Prior studies establish that geometric discrepancies exceeding 1 mm between segmented and true aneurysm morphology correlate with increased intraoperative complications (7), underscoring the need for submillimeter precision in real-world embolization workflows. This demand is particularly acute for small IAs (< 3*mm* diameter), which exhibit higher misclassification rates due to limited contrast retention and overlapping vasculature (10). While recent consensus guidelines highlight IA segmentation as critical for minimizing perioperative risks (5), conventional approaches relying on manual delineation or threshold-based techniques lack the requisite spatial fidelity and speed for intraoperative adoption.

### 2.2 Limitations of current computational approaches

Deep learning has shown promise in addressing medical image segmentation challenges, but existing IA-related studies exhibit clear modality biases (16, 17). For DSA-based approaches, early efforts include Jerman et al.'s work (18), which computed intravascular distance maps from 3D-DSA images for CNN-based classification but suffered from high computational overhead. Podgorsak et al. (19, 20) modified VGG networks to achieve three-class segmentation (background, vasculature, and aneurysm) in DSA with good agreement between predictions and ground truth. Duan et al. (21) proposed a two-stage CNN architecture integrating frontal and lateral views with false-positive suppression to improve detection specificity. For 2D+time DSA sequences, Jin et al. (22) developed a U-shaped network incorporating spatiotemporal information, achieving 89.3% sensitivity but with 3.77 average false positives per sequence, while Liao et al. (23) combined CNNs with ConvLSTM to capture temporal dynamics, enhancing accuracy through multi-frame fusion.

Meanwhile, numerous studies focus on CTA/MRA or 3D rotational angiography (3D-RA) (24, 25). Zhang et al. (26) proposed FSTIF-UNet with feature fusion and attention mechanisms, showing improved performance on 3D-RA for complex vasculature and small aneurysms, though 3D-RA requires multi-angle acquisitions with 17.6-22.2 second processing delays (27–29), rendering it unsuitable for real-time guidance. CTA-specific models (9, 30–32) demonstrate robust detection but are tailored to CT imaging characteristics.

Despite these advancements, critical limitations persist. Conventional 2D DSA segmentation algorithms degrade significantly when vascular overlap exceeds 50% (33), and most deep learning approaches—including DSA-specific ones—exhibit poor performance on small IAs, which constitute over 30% of multi-center cases (21, 23, 34) and whose misidentification impacts rupture risk assessment. Additionally, many studies suffer from limited dataset size, narrow aneurysm type diversity, and sensitivity to DSA image quality variations caused by contrast injection parameters.

Existing deep learning solutions for IA analysis prioritize modalities like CTA and MRA (13, 35), despite DSA's established role as the gold standard for vascular characterization. Methods designed specifically for DSA face three interrelated constraints:

- Temporal Incompatibility: 3D rotational angiography achieves moderate segmentation accuracy (Dice: 0.87–0.90) but requires multi-angle acquisitions incompatible with single-frame navigation needs (27).
- Contextual Blind Spots: Hybrid frameworks (36) integrate symbolic rules with deep networks but fail to model hemodynamic context in overlapping vessels, leading to false positives in >40% of small IAs.
- Morphological Rigidity: Graph-based refinements (37) improve boundary delineation yet remain sensitive to aneurysm shape irregularity—a key predictor of rupture risk neglected in current benchmarks.

### 2.3 Insights from non-DSA vascular segmentation and DSA-specific challenges

Insights from non-DSA vascular segmentation suggest promising avenues for innovation. The integration of learnable edge detectors with attention mechanisms (38) demonstrates enhanced sensitivity to tubular structures in retinal imaging, while dual-path feature fusion (39) mitigates noise in low-dose CT angiography. However, these approaches lack optimization for DSA's projective geometry, where depth ambiguity amplifies topological complexity. Similarly, unsupervised shape modeling techniques (31) reduce annotation dependency but cannot resolve the signal-to-noise limitations inherent in DSA's subtraction artifacts.

## 3 Materials and methods

### 3.1 Dataset collection and preprocessing

**Figure 1:**
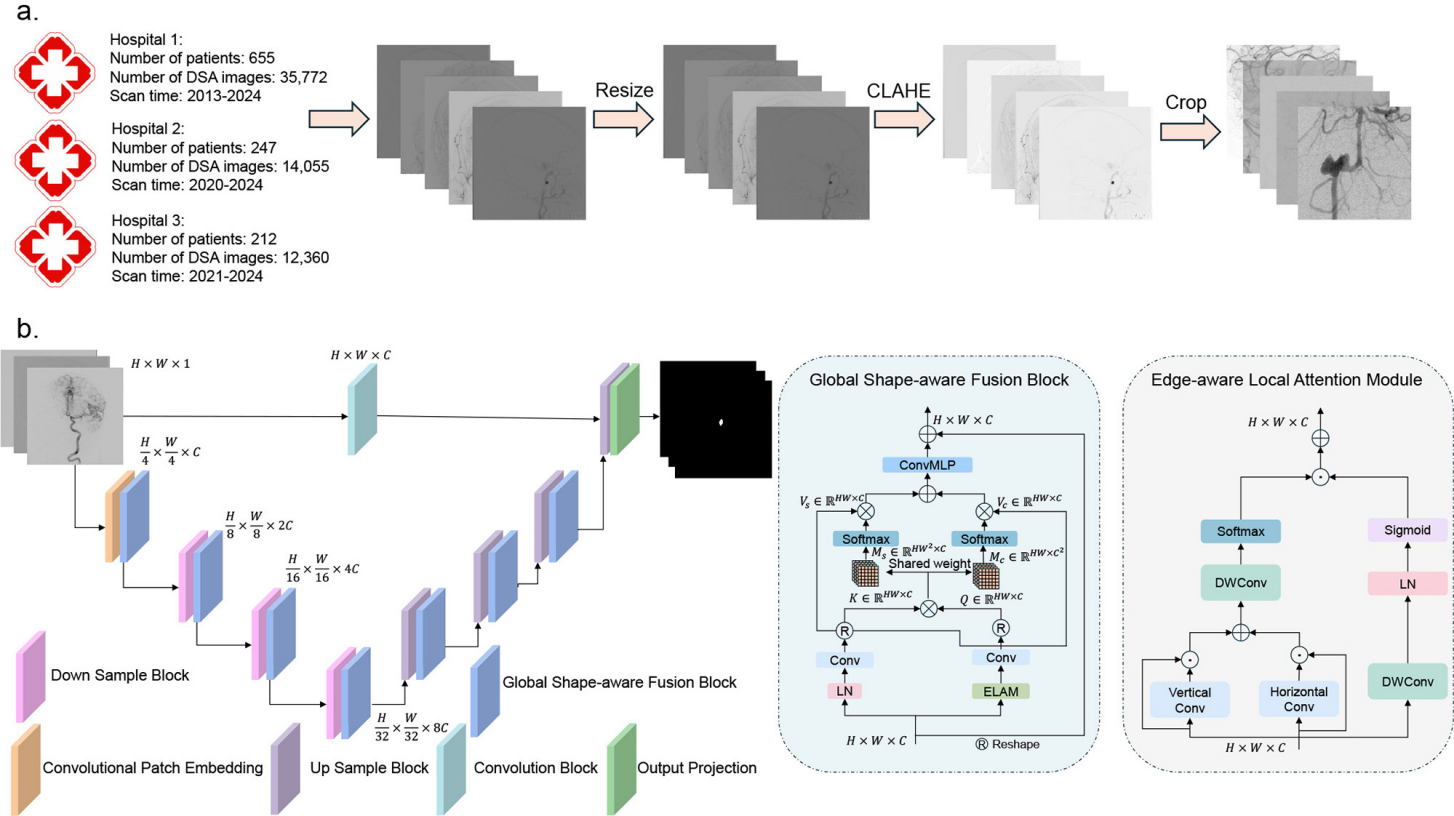
An overview of the pipeline of our paper. **(a)** Data collection and pre-processing in our study. **(b)** The structure of SDAN and the detail structures of GSFB and ELAM. Convolutional Patch Embedding is a overlap patch method with convolution.

This study retrospectively collected 62,187 DSA images from three medical centers between 2013 and 2024. The dataset included: Northern Theater Command General Hospital (Institution 1: 35,772 images from 655 patients), 242 hospital affiliated to Shenyang Medical College (Institution 2: 14,055 images from 247 patients) and the Fourth Affiliated Hospital of China Medical University (Institution 3: 12,360 images from 212 patients). All images were acquired using 300mgI/mL iodinated contrast agent (Schering AG) with a spatial resolution of 0.308 × 0.308 mm and matrix sizes ranging from 960 × 960 to 1, 240 × 1, 240 pixels. The details of data collection and preprocessing is illustrated in Figure 1. Characteristics of the dataset are listed in Table 1. We divided data from Institution 1 into training set and internal test set with a ratio of 8:2, while data from Institution 2 and Institution 3 are used as external test sets. The mean age of the patient was 57.65 years (interquartile range [IQR]: 42–86 years), with balanced sex distribution (56.53% female) and the aneurysms demonstrated a diameter skewness (24.37% small aneurysms < 3*mm*, 9.65% large aneurysms >10*mm*) distributed across critical vascular territories, including the internal carotid artery (17.72%) and the middle cerebral artery (20.25%) (*P* < 0.001). The equipment models are listed in Table 2. To address multicenter scanner variations, at the preprocessing stage, for each image from DSA sequences, we first utilized CLAHE to improve the contrast of vascular structure. Subsequently, we resized images to 512 × 512 to unify the shape of all images.

**Table 1:** Basic information and statistical analysis of the dataset.

**Characteristics**	**Training and internal test set**		**External test sets**		***P* Value^‡^**
	**Training set**	**Internal test set**	**External test set 1**	**External test set 2**	
Patients	536	119	247	212	
Age (y)^*^	57.65 (42–84)	55.69 (41–76)	57.70 (41–86)	56.75 (51–75)	0.004
Sex					0.980
Female	303 (56.53%)	78 (58.21%)	142 (57.49%)	120 (56.60%)	
Male	233 (43.47%)	56 (41.79%)	105 (42.51%)	92 (43.40%)	
DSA equipment	2	2	1	1	
DSA images	28,471	7,301	14,055	12,360	
Aneurysms	632	131	285	237	
Aneurysm size					<0.001
> 10mm	61 (9.65%)	14 (10.69%)	8 (2.81%)	8 (3.38%)	
5 to < 10mm	97 (15.35%)	29 (22.14)	61 (21.40%)	62 (26.16%)	
3 to < 5 mm	320 (50.63%)	40 (30.53%)	101 (35.44%)	92 (38.82%)	
< 3 mm	154 (24.37%)	48 (36.64%)	115 (40.35%)	75 (31.64%)	
Aneurysm location					<0.001
Internal carotid artery	112 (17.72%)	25 (19.08%)	71 (24.91%)	41 (17.30%)	
Middle cerebral artery	128 (20.25%)	23 (17.56%)	37 (12.98%)	29 (12.24%)	
Anterior cerebral artery	25 (3.96%)	6 (4.58%)	7 (2.46%)	11 (4.64%)	
Anterior communicating artery	124 (19.62%)	15 (11.45%)	51 (17.89%)	38 (16.03%)	
Posterior communicating artery	111 (17.56%)	18 (13.74%)	60 (21.05%)	62 (26.16%)	
Posterior cerebral artery	22 (3.48%)	9 (6.87%)	15 (5.26%)	12 (5.06%)	
Vertebral artery	45 (7.12%)	11 (8.40%)	16 (5.61%)	20 (8.44%)	
Basilar artery	35 (5.54%)	10 (7.63%)	7 (2.46%)	11 (4.64%)	
Other^†^	30 (4.75%)	14 (10.69%)	21 (7.38%)	13 (5.49%)	

^*^Data are mean value; data in parentheses are IQRs.

^†^Other locations including pericallosal artery, Anterior cerebellar artery, Posterior cerebellar artery, and Anterior choroid aneurysm.

^‡^Data are considered statistically significant if *P* ≤ 0.05. *P* values were calculated using Mann-Whitney U rank test for continuous distributions; discrete distributions were assessed using Kruskal-Wallis H-test.

**Table 2:** List of equipment models.

**Institution**	**Equipment model**
Institution 1	SIEMENS AXIOM Artis system, Philips Azurion 5M20
Institution 2	SIEMENS AXIOM Artis system
Institution 3	GE MEDICAL SYSTEMS

### 3.2 Research strategy and model architecture

Collectively, prior work reveals a persistent challenge: No framework simultaneously addresses the spatial-temporal constraints of DSA (single frame processing), morphological variability, and clinical deployability. To address this challenge, we developed the Shape-aware Dual-stream Attention Network (SDAN). The design of the network is based on observations of neurointerventional decision making, where clinicians combine local edge discontinuities, such as aneurysm neck morphology, with a global hemodynamic context, including the trajectory of the parent vessel, to determine the location of an intracranial aneurysm. The structure of SDAN is shown in Figure 1b, which incorporates a convolutional patch embedding module as the initial feature extraction step (40). This module replaces traditional patch embedding with task-oriented convolutional operations to reduce computational overhead. This embedding method retains more local image information, thereby improving the effectiveness of feature extraction. Notably, the first feature map undergoes 4× downsampling (rather than 2×) to balance spatial resolution retention with computational efficiency. Given the high native resolution of the DSA images, scaling 4× compresses redundant background information while preserving critical morphological details of small aneurysms and vessel edges. Subsequently, two main modules are designed: Edge-aware Local Attention Module (ELAM) and Global Shape-aware Fusion Block (GSFB). Detailed architectural configurations and functional mechanisms of these components will be elaborated in the subsequent sections.

### 3.3 Edge-aware local attention module

Since human blood vessels are topologically oriented, we transform the gradient computation principle of the classical Canny edge detection operator into a learnable convolutional form to extract anisotropic features of the vascular structure:


(1) $$\begin{array}{l} F_{h}=K_{h}\cdot X, \end{array}$$

(2) $$\begin{array}{l} F_{v}=K_{v}\cdot X, \end{array}$$

where *K*_*h*_ and *K*_*v*_ are the horizontal and vertical convolution kernels with kernel size of 3 × 3, respectively. The boundary enhancement mechanism of the visual system is then simulated using matrix addition:


(3) $$\begin{array}{l} F=F_{h}+F_{v}. \end{array}$$

And the spatial attention weights are generated by depth-separable convolution D, where the Sigmoid function σ is able to constrain the weight range to [0, 1]:


(4) $$\begin{array}{l} A=\sigma \left(D\left(F\right)\right), \end{array}$$

Meanwhile, *X* is processed through D and Layer Normalization (LN) to obtain the channel-related features *F*_*c*_. Then A is weighted onto *F*_*c*_ to obtain the result features:


(5) $$\begin{array}{l} F_{c}=LN\left(D\left(X\right)+X\right), \end{array}$$

(6) $$\begin{array}{l} F_{ELAM}=F_{c}⊙A+X, \end{array}$$

here, ⊙ denotes element-wise multiplication. The design enables ELAM to adaptively focus on morphological discontinuites in the vessel wall, overcoming the shortcoming of traditional fixation operators that are sensitive to biological tissue variability.

### 3.4 Global shape-aware fusion block

Since ELAM is a form of local attention, it causes the network to focus only on features within the receptive field, making it highly sensitive to noise and subtle changes in vascular structures (41, 42). Therefore, we introduced Global Shape-aware Fusion Block (GSFB), a global attention mechanism that combines spatial and channel dimensions. It can map local attention into global attention, reducing sensitivity of the model to noise and enhancing its robustness to slight changes in vascular structures, thereby enabling the acquisition of aneurysm features, as illustrated in Figure 1b. GSFB consists of spatial global attention and channel global attention, utilizing Convolutional Multilayer Perceptron (ConvMLP) to mix these features. First, the output of ELAM (*F*_*ELAM*_), is utilized as query (*Q*), which contains localized shape features. The key (*K*), spatial value (*V*_*s*_), and channel value (*V*_*c*_) are extracted from the input features, ensuring the consistency and stability of the features. In particular, weight sharing between *Q* and *K* is used to reduce computational complexity while maintaining feature alignment. Second, spatial and channel dimensional multi-head attention are processed separately:


(7) $$\begin{array}{l} MHA_{s}=softmax\left(\frac{QK^{T}}{\sqrt{d_{k}}}V_{s}\right), \end{array}$$

(8) $$\begin{array}{l} MHA_{c}=softmax\left(\frac{QK^{T}}{\sqrt{d_{k}}}V_{c}\right), \end{array}$$

where *d*_*k*_ is the key dimension and MHA means multi-head attention. For spatial attention, the computation focuses on inter-region dependencies, while channel attention emphasizes feature channel relationships. This approach enables GSFB to capture both global structural patterns and fine-grained local features. Finally, the output features from spatial and channel branches are mixed through a ConvMLP:


(9) $$\begin{array}{l} F_{fusion}=MHA_{s}+MHA_{c}+LN\left(X\right), \end{array}$$

(10) $$\begin{array}{l} F_{out}=F_{fusion}+ConvMLP\left(F_{fusion}\right)+X, \end{array}$$

where ConvMLP is defined as two convolutions connected with ReLU function, dynamically fuses multi-scale features and suppresses noise by leveraging global context.

Firstly, by extending receptive fields to the entire feature map, GSFB mitigates the over-sensitivity of ELAM to local noise, a limitation observed in traditional local attention mechanisms. Secondly, dual-stream attention ensures sensitivity to vascular deformations and maintain robustness to the diverse shapes of vascular structures, enabling the network to focus on extracting the features of IAs. Thirdly, Weight sharing and parallel spatial-channel processing reduce computational overhead, aligning with the efficiency goals of modern attention architectures (43). The refined architecture balances computational efficiency with medical image analysis tasks especially for specific structures.

### 3.5 Loss function

ELAM and GSFB leverage edge-aware features to improve the ability to identify aneurysms. We seek to augment regional discriminative power of the network with an appropriate loss function design, specifically to dampen its responsiveness to noise and irrelevant edge signals. Thus, we formulate the final loss function as a combination of the Dice and focal losses:


(11) $$\begin{array}{l} L=1-\frac{2|y\cap \hat{y}|}{\left|y|+|\hat{y}\right|}-{\left(1-\hat{y}\right)}^{2}\log\left(\hat{y}\right), \end{array}$$

where *y* is the true label and ŷ is the prediction result.

## 4 Experiment and results

### 4.1 Training and implementation details

In this section, we will introduce the parameters and the implementation details to make sure the reproducibility of SDAN. For the architecture of SDAN, the number of channels in the first layer is 32, and the number of channels in each subsequent layer is twice the number of channels in the previous layer. In addition, the number of channels of skip-connection in the input layer is also 32. Images in training dataset were patched to 224 × 224, while test images were only normalized by CLAHE and the model would predict images by sliding window method, as illustrated in Figure 1a. During the training phase of SDAN, we employed AdamW (44) optimizer with an initial learning rate of 0.0001 to train the model. Besides that, a cosine annealing learning rate schedule was used to decay the learning rate during training. And we set batch size of 8. Our framework is trained and implemented in PyTorch 2.1.1 using a deep learning workstation with two NVIDIA RTX A6000. The detail of hardware and software is illustrated in Table 3.

**Table 3:** Hardware and software environment.

**Name**	**Version**
PC System	Ubuntu 24.04 LTS
CPU	Intel(R) Core(TM) i9-14900K
GPU	NVIDIA RTX A6000 × 2
python	3.10.13
pytorch	2.1.1
monai	1.3.0
timm	0.9.16

### 4.2 Evaluation details

We evaluated SDAN and conducted a detailed comparison with some existing models. These models are as follows: UNet (45), UNetV2 (46), Attention-UNet (47), nnUNet (48), SegNet (49), UNETR (50), SwinUNet (51), SwinUNetR (52), VMUNet (53), VMUNetV2 (54), and HTC-Net (39). UNet and other UNet-like models are used to explain the prior performance for GSFB. In particular, Attention-UNet was utilized for IA segmentation in previous research, and HTC-Net was used to balance local and global information. For all methods, we adjusted the hyperparameters to ensure that the optimal results were obtained during the training process.

The performance of SDAN in the IA segmentation task is evaluated by Dice Similarity Coefficient (DSC), 95% Hausdorff Distance (HD95), Sensitivity, Precision, and Intersection over Union (IoU). A paired *t*-test *P* value less than 0.05 is considered to indicate a statistically difference. Specifically, DSC measures the overlap between the prediction and the ground truth, which is particularly suitable for evaluating small target segmentation tasks. The calculation method is:


(12) $$\begin{array}{l} DSC=\frac{2\cdot |A\cap B|}{\left|A|+|B\right|} \end{array}$$

where A represents the prediction and B represents the ground truth. HD95 quantifies the maximum distance between the prediction boundary points and the ground truth, focusing on evaluating the precision of the segmentation boundaries. It is a robust variant of the standard Hausdorff distance, excluding the top 5% of extreme distance values to reduce the impact of outliers. HD can be calculated as follows:


(13) $$\begin{array}{l} HD=\max\left(\underset{a\in A}{\max}\underset{b\in B}{\min}∥a-b∥,\underset{b\in B}{\max}\underset{a\in A}{\min}∥b-a∥\right) \end{array}$$

HD95 thereby can be calculated by selecting the top 95% of the directed distance values.

In addition, sensitivity reflects the model's ability to correctly identify all true target regions, precision measures the proportion of pixels predicted as target regions that are actually true targets, and IoU evaluates the overlap between the prediction and the ground truth by the ratio of their intersection to their union, providing a stricter measure of overlap compared to DSC. These metrics are calculated as follows:


(14) $$\begin{array}{l} Sensitivity=\frac{TP}{TP+FN} \end{array}$$

(15) $$\begin{array}{l} Precision=\frac{TP}{TP+FP} \end{array}$$

(16) $$\begin{array}{l} IoU=\frac{\left|A\cap B\right|}{\left|A\cup B\right|} \end{array}$$

### 4.3 Model performance on internal test dataset

SDAN was evaluated on 7,301 DSA images from internal test dataset. The results are listed in Table 4. The model achieved a DSC of 0.951, surpassing existing methods by 2.83%–23.54%. The HD95 of SDAN reached 1.995, indicating that the contour of SDAN's results is very close to the contour of the ground truth. SDAN also showed the best performance in Sensitivity, Precision, and IoU, indicating that our model has excellent robustness to noise and a high ability to distinguish IAs from adjacent blood vessels. Evidently, SDAN exhibits superior and more concentrated DSC values, signifying it has a better ability to segment the aneurysm region. Moreover, the more outstanding and clustered HD95 metrics demonstrate SDAN's sensitivity to aneurysm contours, along with its superior discriminatory power between aneurysms and adjacent vascular structures.

**Table 4:** Model performance on internal test dataset with DSC, HD95, Sensitivity, Precision, and IoU.

	**DSC**	**HD95**	**Sensitivity**	**Precision**	**IoU**
UNet	0.821	39.249	0.826	0.845	0.745
UNetV2	0.789	9.226	0.754	0.887	0.710
Attention-UNet	0.855	11.981	0.852	0.894	0.792
nnUNet	0.923	2.365	0.931	0.917	0.875
SegNet	0.848	7.995	0.842	0.891	0.783
UNETR	0.716	109.161	0.732	0.739	0.624
SwinUNet	0.726	15.598	0.733	0.779	0.650
SwinUNetR	0.918	53.164	0.940	0.909	0.860
VMUNet	0.857	10.040	0.853	0.885	0.790
VMUNetV2	0.786	11.519	0.769	0.855	0.703
HTC-Net	0.803	11.072	0.786	0.871	0.727
SDAN	0.951	1.995^*^	0.946^*^	0.959^*^	0.908^*^

The best results are displayed in red, and the second-best results are displayed in blue. ^*^P values indicate significance compared with nnUNet (*P* < 0.05).

### 4.4 Model performance on external test dataset

In this section, we evaluated SDAN using our external evaluation datasets. The total results are illustrated in Table 5. Compared with the results of the internal test dataset, our SDAN performed well in the external test datasets and showed consistency with its performance in the internal test dataset. In terms of overall performance, the HD95 of the external test datasets increased by 0.122 and 0.579, while the Precision results improved by 0.017 to 0.02. For results across different diameters, the external test datasets showed small improvements in small and middle IAs (DSC increased by approximately 0.01). Its performance in large and huge IAs was inferior to that in the internal test dataset, with the DSC value decreasing by approximately 0.015, within the error range, SDAN demonstrated a high degree of consistency in performance across all datasets.

**Table 5:** Model performance on external test dataset with DSC, HD95, Sensitivity, Precision, and IoU.

**Institution**	**DSC**	**HD95**	**Sensitivity**	**Precision**	**IoU**
Institution 1	0.951	1.995	0.946	0.959	0.908
Institution 2	0.943	1.873	0.913	0.976	0.892
Institution 3	0.944	1.416	0.913	0.979	0.895

### 4.5 Ablation studies

We conducted ablation experiments on SDAN to discuss the effects of the modules in SDAN. Firstly, we replaced ELAM with residual block (ResBlock) as baseline model. Secondly, we used only ELAM as the backbone to show the effect of ELAM. Thirdly, we utilized ResBlock to replace GSFB to illustrate the effect of GSFB in SDAN. Finally, we employed a local attention mechanism, named Squeeze-and-Excitation block (SE) (55) to replace ELAM to show the effect of attention mechanism in ELAM.

The quantitative results are illustrated in Table 6. The baseline model showed passable performance with DSC of 0.764, and HD95 was 65.870. When ELAM was added, all results improved. It indicated that ELAM could effectively enhance the performance with DSC increased to 0.809. However, when ELAM was combined with ResBlock, DSC decreased slightly to 0.804, suggesting that this combination was less effective than using ELAM alone, and GSFB outperformed ResBlock. When combined with ELAM, DSC increased significantly to 0.951, which was superior to other combinations. Furthermore, when SE was combined with GSFB, the DSC was 0.797. Despite some improvement in results, it was still much lower than the combination of ELAM and GSFB.

**Table 6:** Performance of ablation studies.

**Baseline**	**ResBlock**	**ELAM**	**SE**	**GSFB**	**DSC**	**HD95**	**Sensitivity**	**Precision**	**IoU**
✓					0.764	65.870	0.782	0.789	0.677
✓		✓			0.809	21.021	0.813	0.835	0.731
✓	✓	✓			0.804	24.436	0.808	0.833	0.727
✓			✓	✓	0.797	27.979	0.811	0.811	0.719
✓		✓		✓	0.951	1.995	0.946	0.959	0.908

The best results are shown in red.

**Figure 2:**
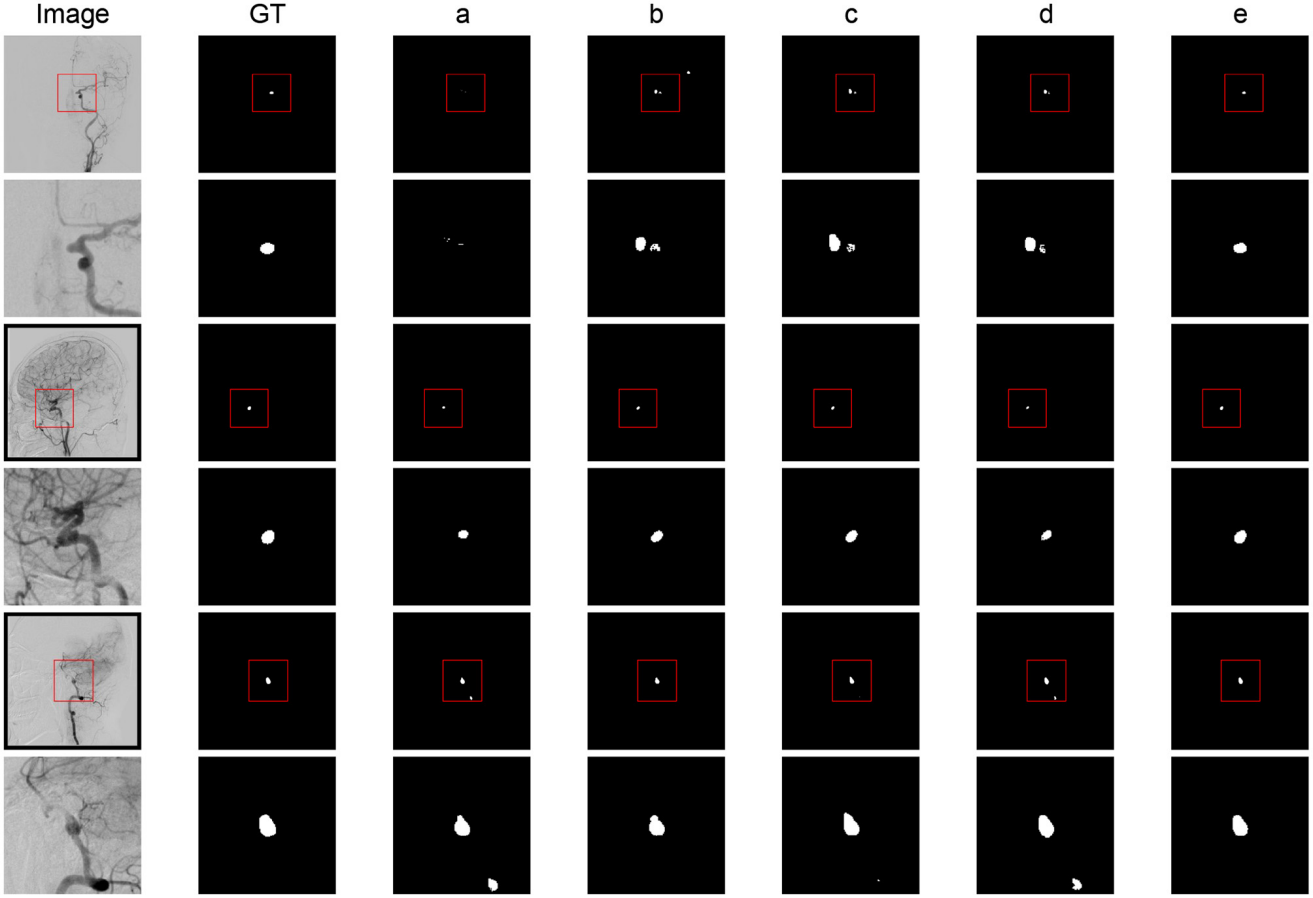
Segmentation results comparison of different modules. **(a)** Baseline model. **(b)** Baseline + ELAM. **(c)** Baseline + ELAM + ResBlock. **(d)** Baseline + SE + GSFB. **(e)** SDAN.

The qualitative results are shown in Figure 2. The baseline models showed a significant deviation from the global truth. The boundaries were unclear with many errors. The result of the combination of baseline and ELAM was obviously improved compared to the baseline model and is closer to the global truth. The segmentation effect of the combination of ELAM and ResBlock was inferior to that of the use of ELAM alone. The effect of SE was also not as good as that of ELAM.

In general, while ELAM improves aneurysm segmentation performance, it exhibits low contour sensitivity and limited discrimination between aneurysms and adjacent vessels. In contrast, integrating GSFB significantly enhances this discriminatory capacity. These findings validate ELAM's boundary sensitivity and GSFB's discriminative power, aligning with the functional analysis results.

## 5 Discussion

### 5.1 Performance of different size

**Figure 3:**
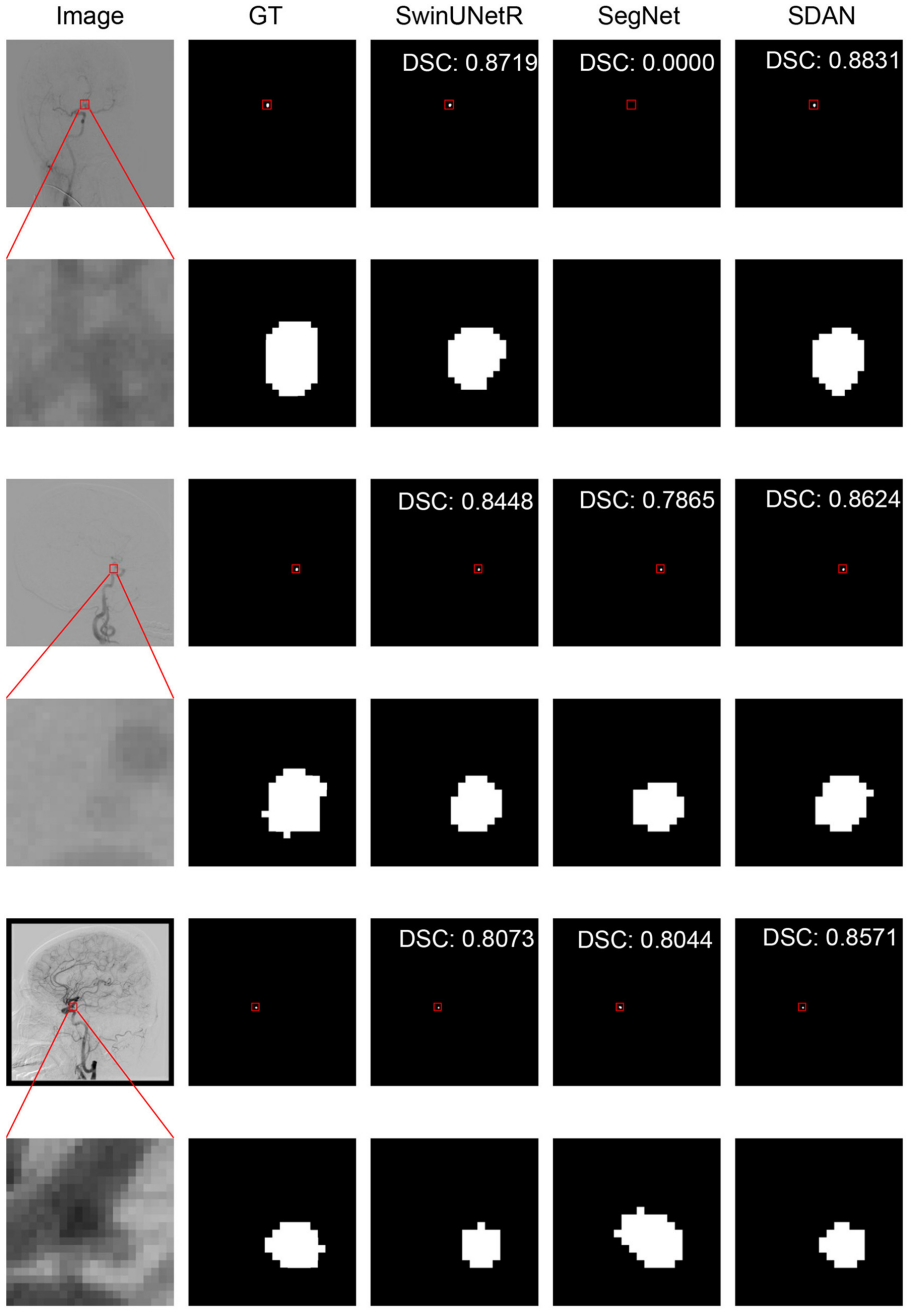
Visual performance on small IAs. The image in the rectangle is at bottom of the image.

**Figure 4:**
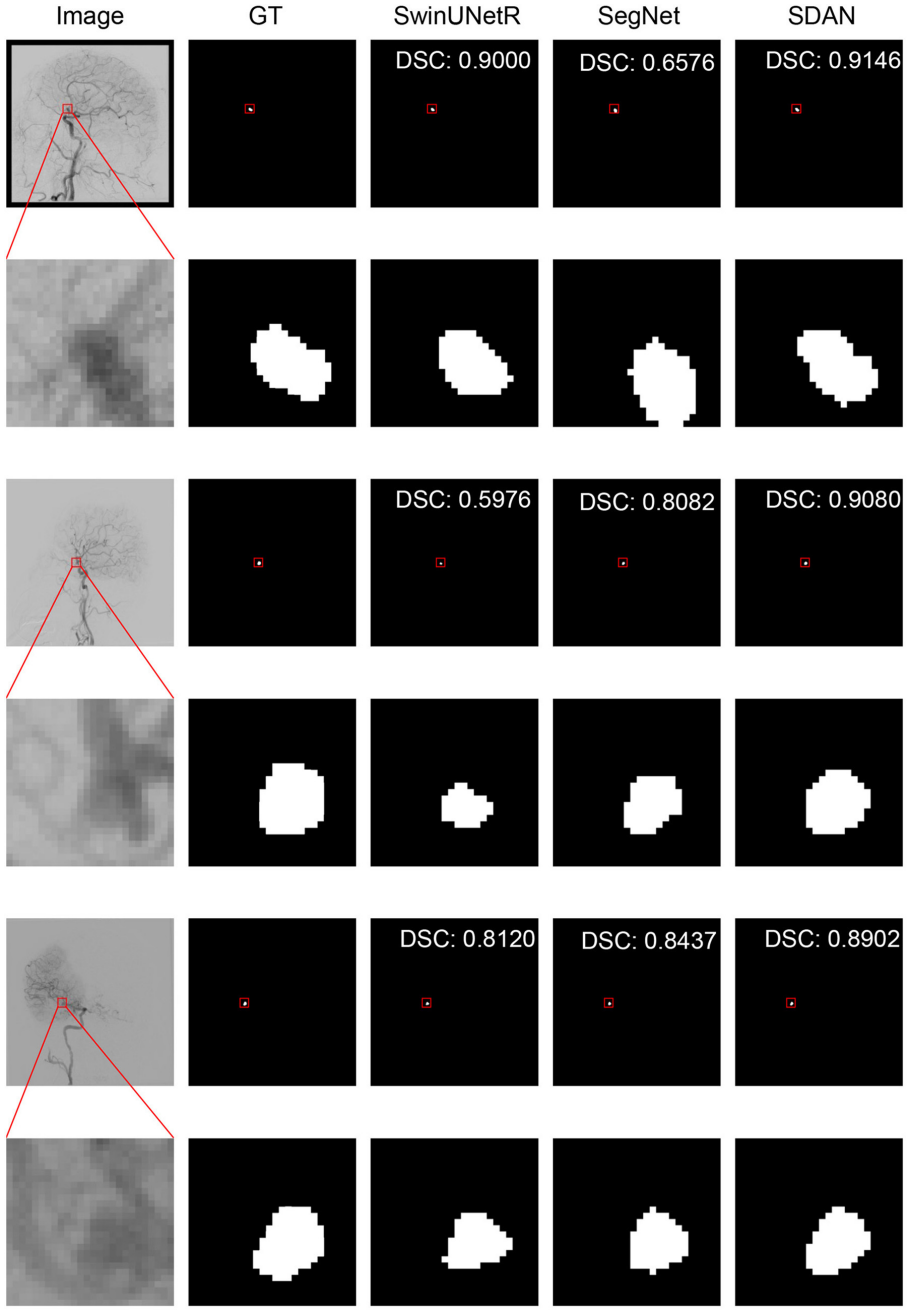
Visual performance on middle IAs. The image in the rectangle is at bottom of the image.

**Figure 5:**
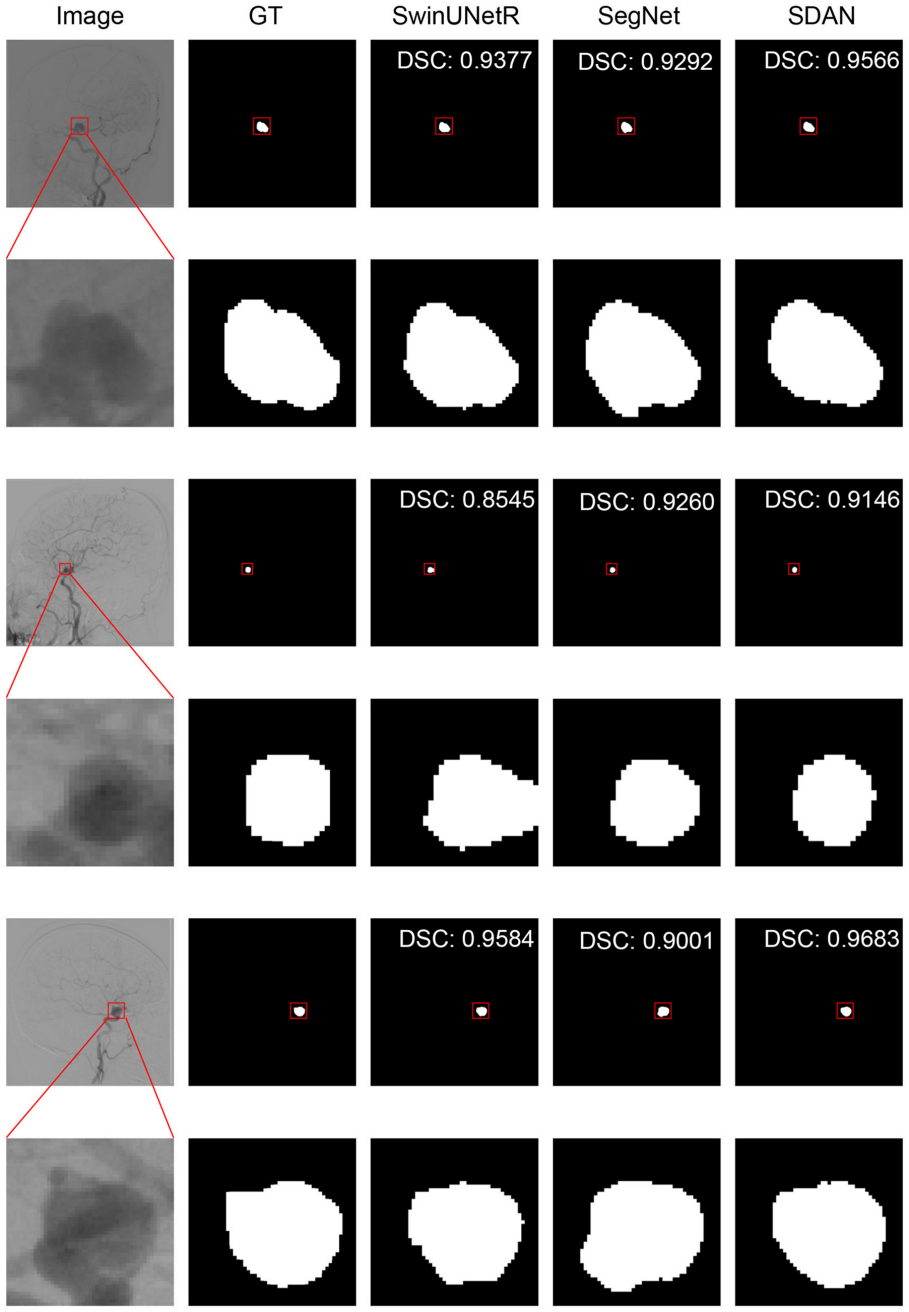
Visual performance on large IAs. The image in the rectangle is at bottom of the image.

**Figure 6:**
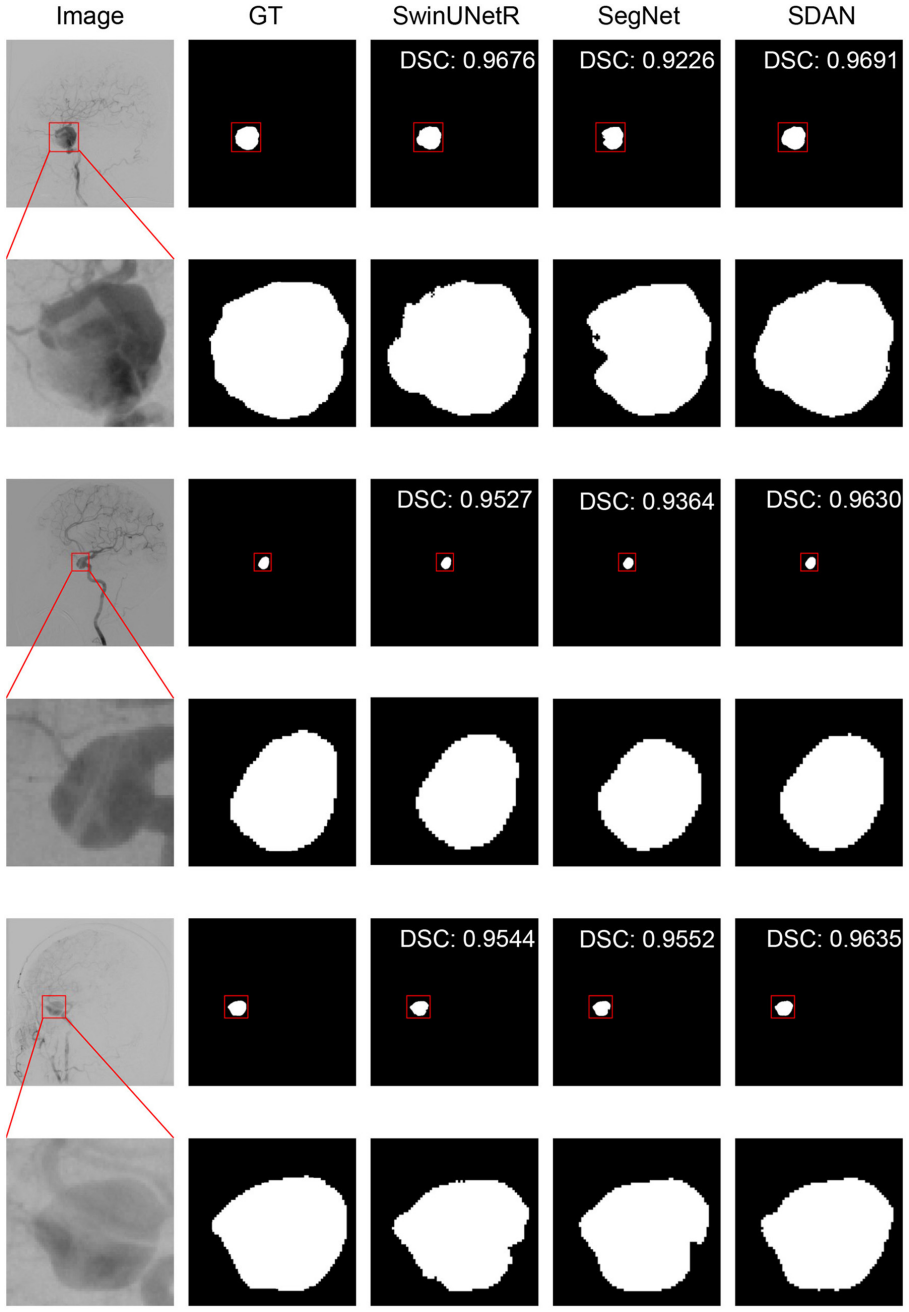
Visual performance on huge IAs. The image in the rectangle is at bottom of the image.

We conducted a detailed analysis of the performance of SDAN for different diameters of IAs. Table 7 systematically presents and compares the results of SDAN and other models under the conditions of multi-scale aneurysm diameters. The results show that SDAN demonstrates excellent stability in the segmentation task of small IAs, with a DSC of 0.929, which is significantly superior to the comparative models by 6.6%–78.4%. Except for nnUNet and SwinUNetR, the remaining methods failed to produce valid results. Although the results of nnUNet are relatively high with a DSC of 0.863, there is still a significant gap compared with SDAN. In the scenario of middle IAs, although the performance of each model has improved compared with that in the group of small IAs, SDAN still remains the best results. When dealing with large and huge IAs, all models show good segmentation results, indicating that the existing methods have reliable processing capabilities for large targets, but generally have shortcomings in segmenting small targets. The visual results are illustrated in Figures 3–6. In these images, we illustrate some common problems for IA segmentation, such as IAs overlapping with blood vessels and IAs with blurred edges. In contrast, SDAN maintains excellent and stable performance across the entire range of aneurysm diameters, without significant accuracy degradation, fully demonstrating its robust adaptability to the size of IAs and its precise segmentation ability.

**Table 7:** Model performance on different sizes of IAs of internal test dataset with DSC, HD95, Sensitivity, Precision, and IoU.

	**DSC**	**HD95**	**Sensitivity**	**Precision**	**IoU**
**Small**					
UNet	0.179	76.036	0.442	0.421	0.283
UNetV2	0.273	11.687	0.227	0.595	0.201
Attention-UNet	0.472	18.552	0.455	0.674	0.404
nnUNet	0.863	9.517	0.873	0.852	0.847
SegNet	0.482	12.444	0.498	0.615	0.410
UNETR	0.229	150.857	0.296	0.242	0.161
SwinUNet	0.145	44.002	0.163	0.354	0.111
SwinUNetR	0.844	70.412	0.891	0.829	0.758
VMUNet	0.548	22.456	0.571	0.626	0.454
VMUNetV2	0.342	23.677	0.366	0.503	0.246
HTC-Net	0.365	17.597	0.391	0.863	0.295
SDAN	0.929	1.546^*^	0.908^*^	0.957^*^	0.869^*^
**Middle**					
UNet	0.693	27.818	0.703	0.739	0.589
UNetV2	0.558	12.183	0.511	0.769	0.454
Attention-UNet	0.756	9.205	0.737	0.822	0.650
nnUNet	0.892	6.221	0.896	0.901	0.866
SegNet	0.699	9.714	0.700	0.784	0.606
UNETR	0.480	90.420	0.486	0.532	0.381
SwinUNet	0.351	28.309	0.399	0.443	0.275
SwinUNetR	0.889	32.383	0.905	0.889	0.810
VMUNet	0.694	14.762	0.705	0.739	0.596
VMUNetV2	0.538	15.390	0.529	0.686	0.433
HTC-Net	0.603	17.317	0.619	0.697	0.507
SDAN	0.935^*^	1.441^*^	0.932^*^	0.940^*^	0.878^*^
**Large**					
UNet	0.866	35.052	0.867	0.885	0.866
UNetV2	0.862	8.899	0.839	0.916	0.862
Attention-UNet	0.891	10.463	0.899	0.907	0.891
nnUNet	0.931	2.147	0.940	0.938	0.897
SegNet	0.899	7.162	0.896	0.924	0.899
UNETR	0.773	104.903	0.800	0.781	0.773
SwinUNet	0.835	10.859	0.847	0.858	0.835
SwinUNetR	0.923	55.297	0.949	0.908	0.923
VMUNet	0.908	8.270	0.904	0.926	0.908
VMUNetV2	0.856	10.967	0.848	0.894	0.856
HTC-Net	0.868	9.439	0.850	0.912	0.848
SDAN	0.954^*^	2.002^*^	0.952^*^	0.958^*^	0.954^*^
**Huge**					
UNet	0.914	43.086	0.911	0.929	0.851
UNetV2	0.911	7.964	0.867	0.967	0.842
Attention-UNet	0.938	14.166	0.927	0.955	0.888
nnUNet	0.943	2.011	0.941	0.946	0.902
SegNet	0.929	7.565	0.910	0.957	0.874
UNETR	0.854	115.485	0.851	0.886	0.764
SwinUNet	0.885	12.902	0.865	0.927	0.807
SwinUNetR	0.942	57.092	0.956	0.934	0.896
VMUNet	0.933	7.918	0.916	0.957	0.879
VMUNetV2	0.906	8.398	0.866	0.960	0.834
HTC-Net	0.907	9.424	0.865	0.966	0.838
SDAN	0.965^*^	2.471 (*p* = 0.12)	0.960^*^	0.971^*^	0.933^*^

^*^P values indicate significance compared with nnUNet (*P* < 0.05). The best results are displayed in red, and the second-best results are displayed in blue.

### 5.2 Functional analysis of modules

**Figure 7:**
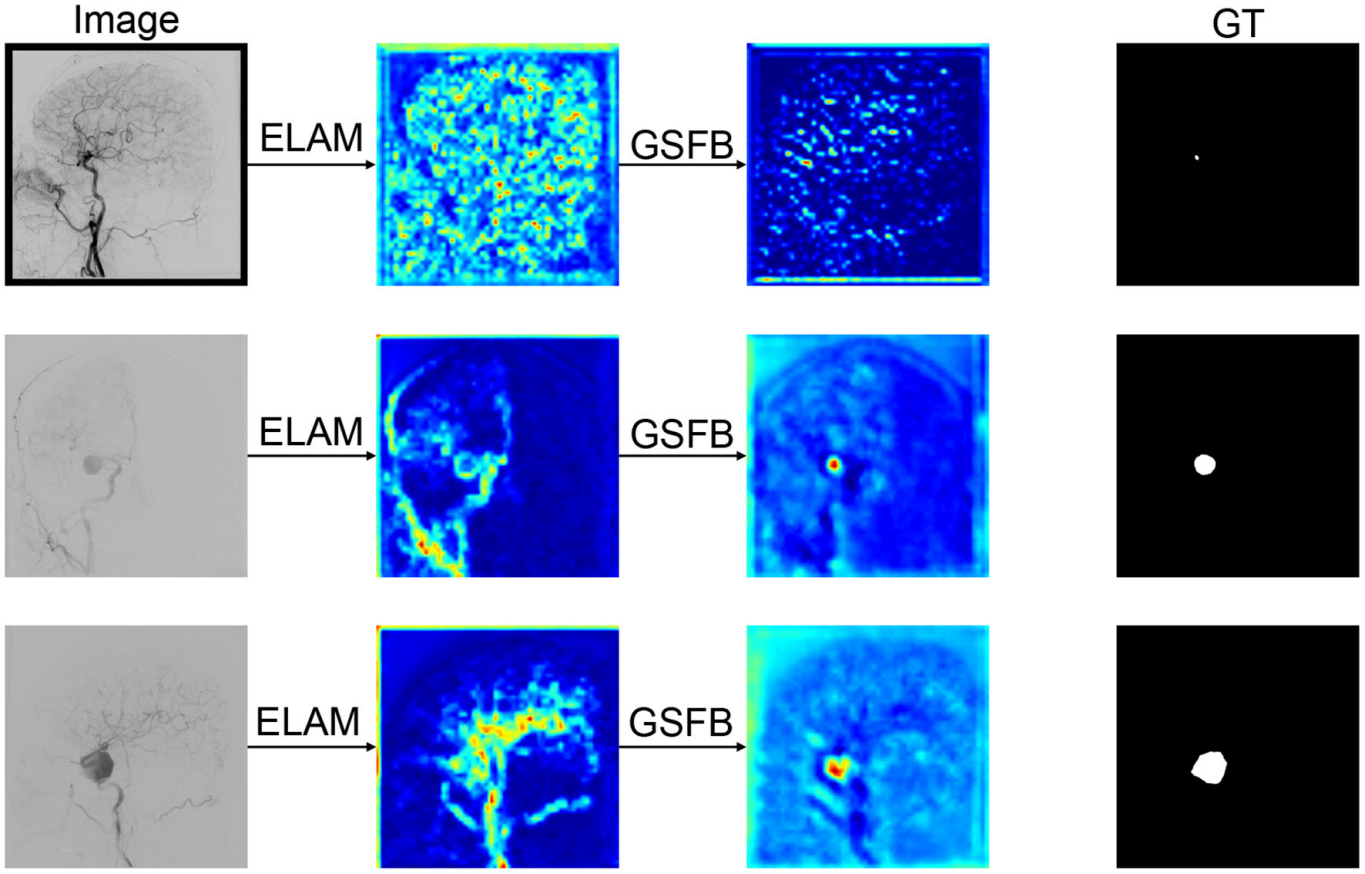
Heatmap of the modules. The images from left to right are input image, the results of ELAM, the results of GSFB, ground truth, respectively.

The heatmap is shown in Figure 7. The heatmap results show that when an image is input into ELAM, the output heatmap demonstrates significant attention to the contours of the vascular structure, but the response to IAs is relatively weak. This phenomenon can be attributed to the fact that ELAM constructs local attention through the collaborative mechanism of horizontal and vertical convolutions, making the module highly sensitive to the boundary features within the local window. However, due to the lack of integration of global semantic information, ELAM has difficulty achieving the differential recognition of IAs and vessels. After being processed by GSFB, there is a significant transformation in the attention distribution of the heatmap: the response intensity in the aneurysm area has been greatly enhanced, while the attention to the contours of vascular structure has decreased significantly. This optimization benefits from the fact that GSFB uses the output features of ELAM as queries and maps them into the global attention mechanism. Under the joint action of spatial and channel dimensions, it can achieve accurate detection of the aneurysm contours and simultaneously suppress interference of vessel characteristics. The relevant evidence will be further demonstrated in the ablation studies.

## 6 Conclusion

In this paper, we introduced a deep learning model named Shape-aware Dual-stream Attention Network (SDAN) for accurate intracranial aneurysm (IA) segmentation from single-frame Digital Subtraction Angiography (DSA). We conducted thorough evaluations using multi-center data. Our experimental results demonstrated that SDAN outperformed all baseline models in all datasets. Crucially, it maintained robust performance, particularly in segmenting small aneurysms. Therefore, this algorithm holds strong potential as an effective auxiliary tool for clinical intracranial aneurysm diagnosis and treatment, thereby enhancing physicians' diagnostic and therapeutic efficiency. Current limitations include slightly reduced performance on external datasets, potentially attributable to variations in image quality across institutions. SDAN is designed based on approaches for small target segmentation (like aneurysm segmentation), and we believe it has the potential to accomplish various small target segmentation tasks. In future work, we will further explore whether this neural network can handle segmentation tasks involving other types or modalities of data.

## Data Availability

The data analyzed in this study is subject to the following licenses/restrictions: the data cannot be made publicly available upon publication because they are owned by a third party and the terms of use prevent public distribution. The data that support the findings of this study are available upon reasonable request from the authors. Requests to access these datasets should be directed to Ma He, mahe@bmie.neu.edu.cn.
